# Intercalation-free, fast switching of mesoporous antimony doped tin oxide with cathodically coloring electrochromic dyes†

**DOI:** 10.1039/d1na00877c

**Published:** 2022-03-17

**Authors:** Jonas Klein, Alexander Hein, Ellen Bold, Fatih Alarslan, Egbert Oesterschulze, Markus Haase

**Affiliations:** Institute of Chemistry of New Materials, University of Osnabrück Barbarastraße 7 D-49076 Osnabrück Germany markus.haase@uni-osnabrueck.de; Department of Physics, Technische Universität Kaiserslautern Erwin-Schrödinger-Str. 46 D-67663 Kaiserslautern Germany

## Abstract

Mesoporous nanoparticle layers of transparent conductive oxides (TCOs) with anchored organic dyes are of great interest for electrochromic applications. Herein, we prepared mesoporous layers of antimony doped tin oxide (ATO) consisting of only 5 nm large particles with a low Sb concentration (2% antimony). The particles were prepared *via* a modified synthesis procedure based on hexahydroxostannate and pure Sb(v) hexahydroxoantimonate(v). We show that the ATO layers benefit from using a non-intercalating electrolyte such as tetrabutylammonium perchlorate (TBAP) compared to lithium perchlorate. Especially in the negative potential range, negative side effects, such as degradation due to lithium intercalation, are reduced. Furthermore, comparing the behavior of particles with varying antimony doping concentrations showed that the particles doped with 2% Sb are most suitable with respect to their conductivity and transparency. When modified with an electrochromic dye (viologen), the hybrid electrodes allow fully reversible (de)coloration with the non-intercalating electrolyte. Similar viologen/TiO_2_ electrodes on the other hand show severely restricted performance with the non-intercalating electrolyte as the oxidation of the dye is partially inhibited. Finally, we built a full electrochromic device composed of two ATO electrodes, each bearing a different electrochromic dye with TBAP as the electrolyte. Despite the dense morphology of the layers due to the small particle size as well as the large size of the electrolyte cation, the device displays remarkable switching times below 0.5 s.

## Abstract 2

SnO_2_:Sb nanoparticles with a small size of 5 nm and a low dopant concentration of 2% allow the fabrication of fast-switching electrochromic devices even with large, non-intercalating cations in the electrolyte.

## Graphical abstract

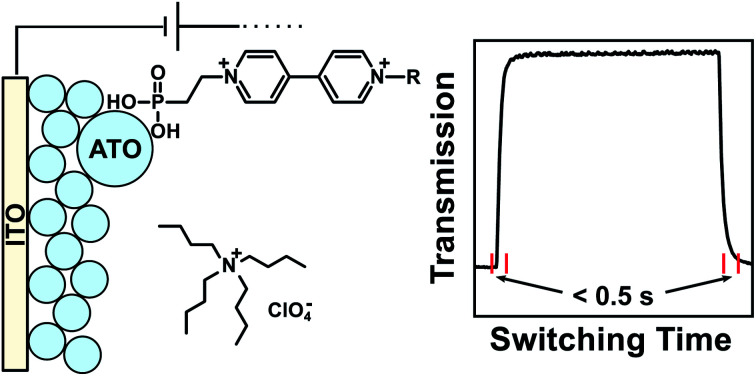

## 1 Introduction

Electrochromic (EC) materials allow for dynamic changes in their absorption behavior regulated by the application of electric fields.^1^ Due to this property such materials are widely investigated for displays,^2,3^ smart windows^4,5^ or imaging.^6,7^ The variety of active materials include metal oxide films,^8,9^ polymer films,^10–13^ solubilized organic molecules^14–16^ or hybrid systems. The latter refers to a combination of an inorganic mesoporous scaffold functionalized with organic redox-active dyes. With respect to the inorganic scaffold, high transparency in the visible region (*i.e.*, low absorption and low scattering of light), as well as good electrical conductivity in the potential range of the redox dye are required. Materials that satisfy these criteria are nanocrystalline transparent conductive oxides (TCOs), which are widely used as scaffold materials. The preparation of a mesoporous scaffold includes doctor blading of a nanoparticle paste of the desired TCO onto a conductive substrate and subsequent calcination. The TCO layer is then functionalized with an organic dye by anchoring the dye molecules to the large inner surface of the mesoporous layer. Standard anchoring groups of the dyes are carboxylate or phosphonate moieties.^17^ Concerning the performance of the dye, an uncolored state with high transparency and a colored state with a large extinction coefficient are desirable. By choosing appropriate materials, such hybrid systems allow for high electrochromic contrasts as well as fast switching times due to fast electron transport throughout the mesoporous film.^18–20^

A class of materials that are widely employed as cathodically coloring dyes are substituted 4,4′-bipyridinium salts commonly known as viologens. Their popularity is based on their facile synthesis, tunable absorption properties, and high stability.^21^ Furthermore, the redox potential of viologens is only slightly negative, which is beneficial with respect to the power consumption of EC devices.^22^ In combination with viologens, titanium dioxide (TiO_2_) is widely applied to form a mesoporous TCO scaffold.^23,24^ TiO_2_ layers offer exceptional transparency in the visible region and TiO_2_ is also readily available.^25^ However, the electrical conductivity of TiO_2_ is restricted to negative potentials unless special electrolytes are chosen allowing the broadening of the potential range.^26^ An example is electrolytes containing small cations, *e.g.* lithium, which are known to shift the conductivity range of TiO_2_ towards more positive potentials. On the downside, using electrolytes with lithium ions can cause lithium insertion in the inorganic scaffold material when the electrodes are sufficiently negatively polarized. Such insertion processes cause mechanical stress within the oxidic nanoparticles due to volume expansion and thereby can lead to performance degradation.^27^

Besides TiO_2_, the combination of other TCOs with viologens has been reported.^28–30^ Among these materials, antimony doped tin oxide (ATO) is very appealing with respect to its spectrum of electrical conductivity. However, in the reports so far, ATO/viologen hybrid layers display inferior performance compared to other TCO/viologen systems,^30^ especially when the nanoparticle size is decreased.^31^ The latter aspect, namely the morphology of the TCO layer, is known to have considerable influence on the performance of such electrochromic systems.^32^ Therefore, ATO layers are primarily employed as counter electrodes in EC devices in the form of ion storage layers^33,34^ or in combination with anodically coloring materials.^35–37^ To improve the performance of ATO/viologens layers, multiple factors have to be taken into account. With ATO being an n-conductive oxide, the charge of the free electrons in the conduction band is compensated for by Sb^5+^ dopant ions occupying Sn^4+^ sites in the crystal lattice.^38^ One potential drawback of ATO is its intrinsic absorbance in the infrared region which extends into the visible region. The strong NIR absorption is caused by the free electrons in the conduction band.^39^ However, as this optical behavior depends on the antimony doping level, a low doping concentration could still offer acceptable transparency while the electrical characteristics are less dependent on the electrolyte composition. Moreover, due to the high mobility of the electrons, comparatively high conductivity of the particles may already be achieved with low antimony concentrations.^40^ A low doping concentration is also known to provide more homogeneous doping, especially when an Sb^5+^ rather than an Sb^3+^ source is used in the synthesis, while increased antimony contents lead to accumulation of antimony at the surface of the particles.^41^ Too high antimony concentrations, however, decrease the conductivity of ATO.^40,42^ This is crucial when small nanoparticles are used to form the mesoporous layers. While small particles offer better optical properties, mesoporous layers composed of small particles are particularly susceptible to increased contact resistance between the nanoparticles. For mesoporous layers, homogeneously doped particles with a low concentration of Sb^5+^ should therefore be beneficial to increase the performance of ATO/viologen systems.

In the present study, we investigate the behavior of a mesoporous ATO layer consisting of small nanoparticles (5 nm) in combination with a viologen. Initially, the spectroelectrochemical response of the bare metal oxide layer is investigated. Since the electrolyte may have a great impact on the performance, an electrolyte containing lithium and a lithium-free electrolyte are used to highlight the benefits when using the latter. Additionally, the spectroelectrochemical performance of particles with different antimony doping concentrations is investigated to choose the optimal Sb content. Comparing the performance of viologen modified ATO and TiO_2_ layers with a non-intercalating electrolyte highlights the superiority of ATO in such a system. Therefore, the viologen functionalized ATO layer is implemented in a sealed two-electrode device. The counter electrode of this device also consists of a mesoporous ATO layer but with an anodically coloring organic dye. The selection of the dyes for the two electrodes is based on our preliminary work^7,18^ and enables the devices to achieve high contrasts. Also in the device, the non-intercalating electrolyte is utilized. In particular, we show that the switching times of the ATO electrodes are quite remarkable, despite the use of electrolytes with large cations and the use of ATO nanocrystals of small size.

## 2 Results and discussion

**Fig. 1:**
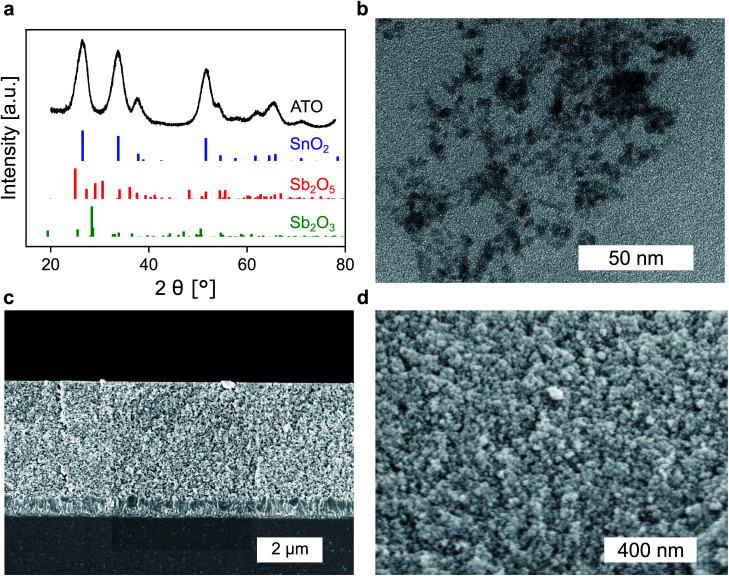
(a) XRD of ATO nanoparticles (2% Sb) along with the reference spectra of SnO_2_ (ICSD: 39177, blue), Sb_2_O_5_ (ICSD: 8050, red) and Sb_2_O_3_ (ICSD: 2033, green). (b) TEM image of ATO nanoparticles after autoclave treatment. (c) Cross-sectional SEM of ATO layers on FTO. (d) Magnified cross-sectional view displaying the mesoporous structure of the nanoparticle layers.

Whether a material is suited for application in an electrochromic device depends on the material's optical and electrochemical properties. Herein the electrochromic system is based on two electrodes each consisting of a mesoporous ATO nanoparticle layer modified with an electrochromic dye. As outlined in the Introduction, a homogeneous distribution of the dopant ions in ATO nanoparticles is achieved with Sb^5+^ ions and low doping concentrations. Thus, a stable Sb^5+^ precursor is required for the synthesis. The widely utilized SbCl_5_ for instance is susceptible to degradation to SbCl_3_. Therefore, we used hexahydroxostannate and hexahydroxoantimonate as precursors and initially added hydrogen peroxide to the hexahydroxoantimonate solution to oxidize all possible present Sb^3+^ ions. Previous reports in which these precursors were used also confirmed high Sb^5+^/Sb^3+^ ratios.^43^ In our case, the nanoparticles were obtained by precipitation through the addition of an acid followed by autoclavation of the nanoparticle colloid as described in the Experimental section. The characterization of the ATO nanoparticles with an antimony doping concentration of 2% was carried out by X-ray powder diffraction (XRD). As shown in Fig. 1a, the particles crystallized in a tetragonal cassiterite structure. There are no peaks corresponding to antimony oxide species such as Sb_2_O_5_ or Sb_2_O_3_. Therefore, successful integration of the dopant ions into the SnO_2_ lattice can be anticipated. According to Rietveld analysis, the particles have a size of around 3.7 nm. The particles were also characterized by transmission electron microscopy (TEM) which also confirms the small size of the nanoparticles (Fig. 1b). However, due to aggregation during drying on the TEM grid, a detailed analysis of sizes from the TEM image is difficult. Dynamic light scattering (DLS) measurements of the colloidal nanoparticles also show average sizes of around 4 nm (Fig. S1, ESI†). With these nanoparticles, a paste was prepared which was then used to fabricate the electrodes by the doctor blade method on fluorine-doped tin oxide coated glass (FTO). After calcination at 450 °C, the ATO layers have a thickness of approx. 3.5 μm as can be estimated from the scanning electron microscopy (SEM) image displayed in Fig. 1c. Calcination also leads to an increase of the particle size to around 5 nm. From the magnified SEM image (Fig. 1d), the mesoporous nature of the layers can be confirmed. Consistent with the small particle size, the ATO layers are very densely packed with small pores. As mentioned in the Introduction, the morphology of mesoporous layers can influence the electrochemical performance and is reported to benefit from larger particles.^31,32^ Especially the contact resistance between the ATO particles has to be considered since their size is very small.

**Fig. 2:**
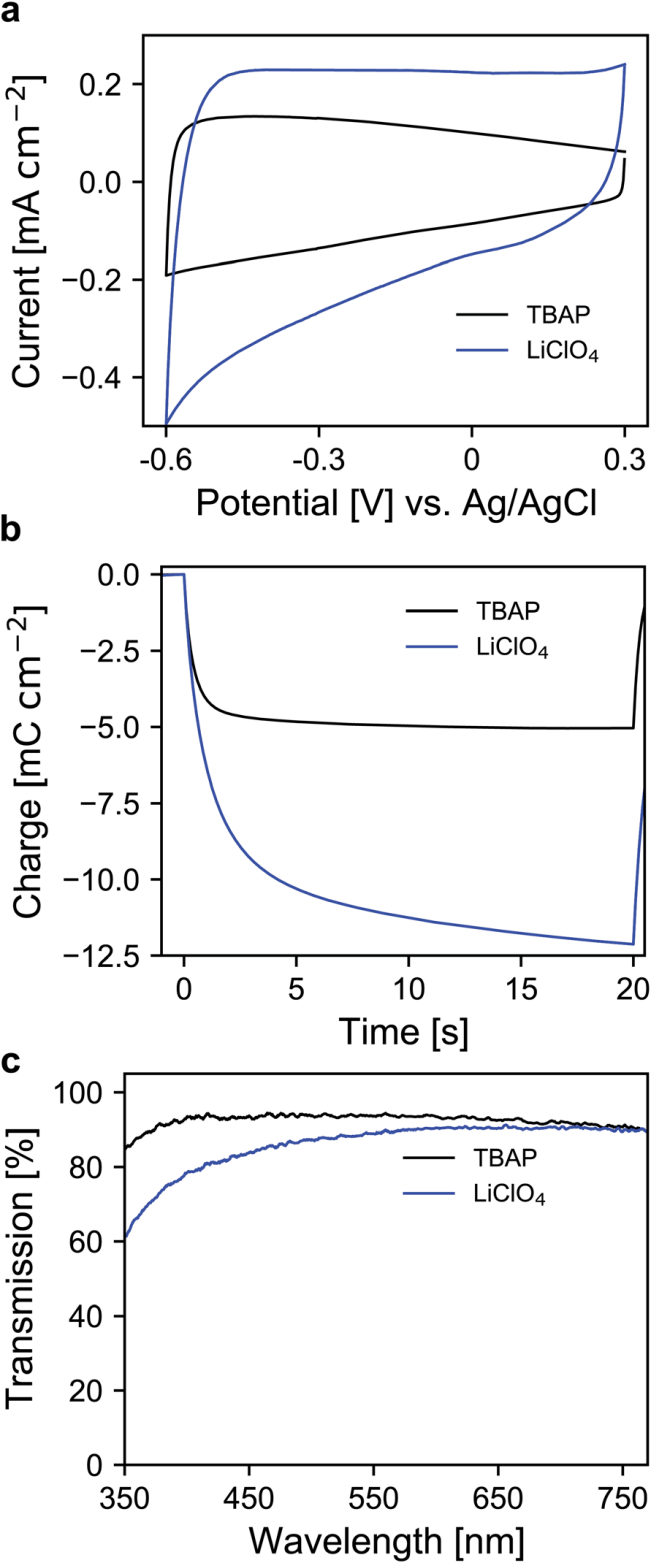
(a) CVs of ATO (2% Sb) mesoporous electrode layers measured in TBAP/PC and LiClO_4_/PC with a scan rate of 20 mV s^−1^. (b) Coulometric measurement for a potential step between +0.3 V and −0.6 V *vs.* Ag/AgCl in both electrolytes. (c) UV/VIS transmission spectra of ATO electrodes (after negative polarization) without an applied potential for both electrolytes; optical reference: an electrolyte-filled cell with blank FTO glass.

Therefore, the electrochemical behavior of the pure ATO layers was initially investigated in a three-electrode setup. Since the performance of the electrodes also depends on the electrolyte composition, the experiments were performed with both 1 M lithium perchlorate (LiClO_4_) and 1 M tetrabutylammonium perchlorate (TBAP) in propylene carbonate (PC) as electrolytes. The corresponding cyclic voltammograms (CVs) are displayed in Fig. 2a. With TBAP/PC as the electrolyte, ATO shows a capacitive current over the whole chosen potential region indicating electron injection into the nanoparticle layer and the formation of an electrochemical double layer.^39^ The current decreases towards positive potentials due to the depletion of electron density at the nanoparticle surface.^44^ With LiClO_4_/PC as the electrolyte, the general characteristics are comparable to that observed with TBAP/PC but with some distinct differences. ATO again shows a capacitive current over the complete potential region. Compared to TBAP, however, the current is significantly higher. This can be explained by the difference in the size of the cations present in the electrolyte and the fact that a tetrabutylammonium ion occupies more space in the capacitive double layer than a lithium ion. Therefore, fewer cations can be incorporated into the double layer when TBAP is used, thereby reducing the number of electrons that can be injected into the ATO layer. The noticeable increase of current at negative potentials as well as the observation that the current towards positive potentials does not drop as sharply as it occurred with TBAP can be assigned to lithium intercalation into the nanoparticle layer. With respect to the long-term performance of such metal oxide layers, lithium intercalation is unfavorable due to the mechanical stress exerted on the nanoparticles due to volume expansion. Especially ATO layers are known to degrade upon lithium insertion.^27^ With TBAP/PC on the other hand, no intercalation phenomena are observed. As already mentioned, the charge consumed by the pure ATO films due to the formation of electrochemical double layers differs significantly based on the electrolyte composition and is further emphasized in a coulometric potential step measurement (Fig. 2b). In the case of the lithium-containing electrolyte, not only is the charge increased by a factor of 2.4 compared to TBAP, but the response time is also slower with LiClO_4_. Along with the electrochemical properties of the pure metal oxide layers, their optical properties were also investigated by means of ultraviolet-visible spectroscopy (UV/VIS). The corresponding transmission spectra of the ATO layers without an applied potential in both electrolytes are displayed in Fig. 2c. Notably, the spectra were recorded after the layers were subjected to polarization at negative potentials. In the case of the TBAP/PC electrolyte, the ATO layer displays high transparency over the complete visible region. In fact, the average transmission of the electrode between 350 and 750 nm reaches 92.6%. With LiClO_4_/PC, the ATO layer suffers from reduced transparency at lower wavelengths after negative polarization, which gives the electrode a permanent yellow coloration. This observation is again a result of lithium intercalation into the nanoparticle layer, which irreversibly degrades the electrode. Therefore, in this case, the average transmission reaches only 86%. From the results presented so far, it can be concluded that the mesoporous ATO layers display better performance with a non-intercalating electrolyte. This is due to the absence of undesirable side effects based on intercalation processes. Furthermore, with respect to the application in electrochromic devices, the charge consumed by the electrode has to be considered since this charge has to be compensated for by the counter electrode in the device. Therefore, utilizing TBAP/PC is again favorable since less charge is consumed. Thus, this electrolyte composition was used in the following experiments.

**Fig. 3:**
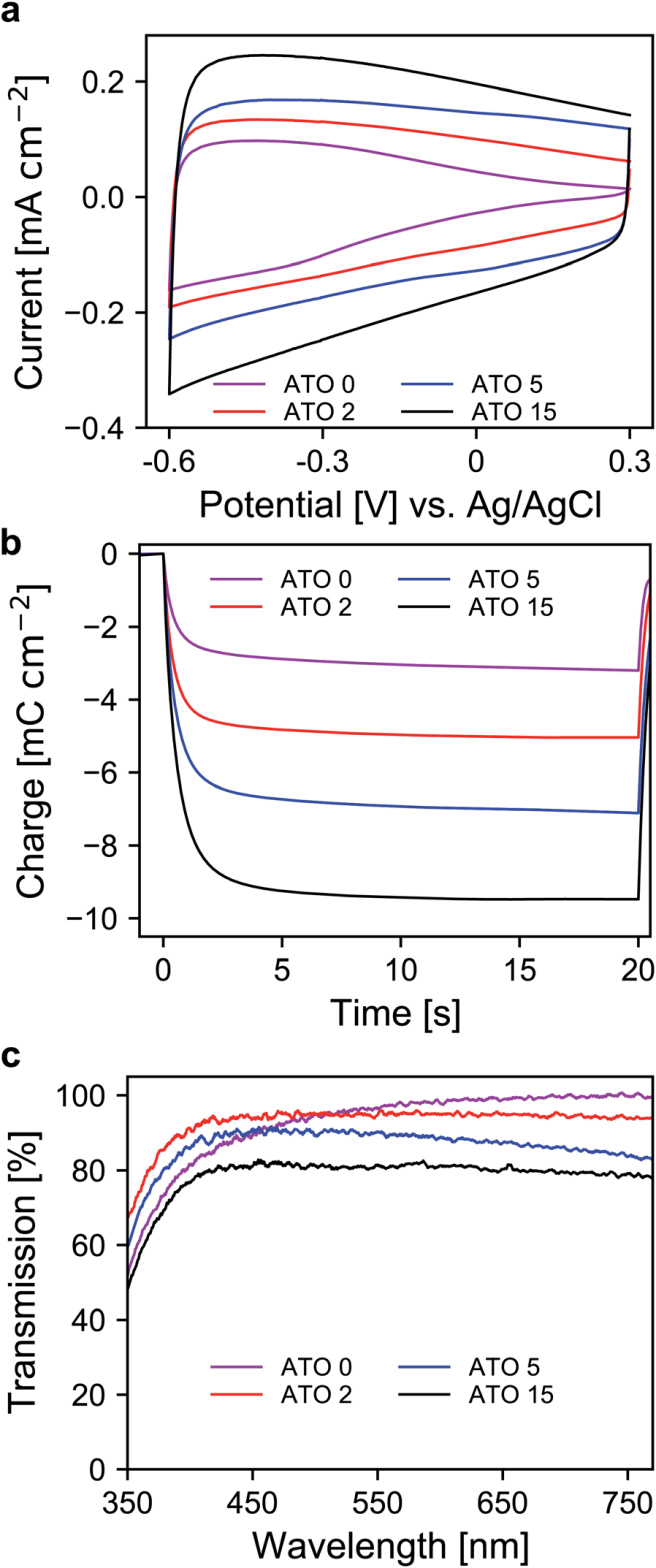
(a) CVs of ATO mesoporous electrode layers with different antimony doping levels measured in TBAP/PC with a scan rate of 20 mV s^−1^. (b) Coulometric measurement for a potential step between +0.3 V and −0.6 V *vs.* Ag/AgCl. (c) UV/VIS transmission spectra of ATO electrodes at +0.3 V *vs.* Ag/AgCl; optical reference: an electrolyte-filled cell with blank FTO glass.

After the selection of a suitable electrolyte, we further investigated the spectroelectrochemical behavior of mesoporous layers of ATO nanoparticles with varying antimony doping concentrations. These were namely undoped SnO_2_ (ATO 0), 2% Sb (ATO 2), 5% Sb (ATO 5) and 15% Sb (ATO 15). The XRD spectra of the nanoparticles are shown in Fig. S2 (ESI†) and confirm that only the pure SnO_2_ phase was obtained. The mean size of the particles slightly increases from approximately 3 to 4.5 nm with decreasing antimony concentration. The CV measurements of the electrodes are displayed in Fig. 3a. The response of all electrodes follows the same general scheme. Particularly, in all cases again a capacitive current can be observed, which decreases towards positive potentials as discussed earlier. The layers with undoped nanoparticles however carry considerably less current at positive potentials, which indicates a limitation with respect to the conductivity of the nanoparticle layer in the respective potential region. All of the doped nanoparticles on the other hand show better conductivity at positive potentials due to the n-doped nature of the nanoparticles. A second observation concerns the differences in the capacitive current of the electrodes. Here, with increasing antimony content the capacitive current also increases as the antimony content influences the number of electrons that can be injected into the nanoparticle layers. This is again also reflected in the charge stored by the bare nanoparticle layers as shown in Fig. 3b. The corresponding values range from around 3 mC for the undoped SnO_2_ to around 11 mC for the particles doped with 15% antimony. Again, this has to be considered with respect to charge compensation by the counter electrode in an electrochromic device. The UV/VIS spectra of the electrodes are presented in Fig. 3c. The undoped nanoparticles show the highest transparency at higher wavelengths. However, towards the UV region, these electrodes suffer from a similar problem to the ATO electrode measured with a lithium-containing electrolyte. This indicates that after negative polarization, charge carriers are irreversibly trapped within the SnO_2_ particle layer, which absorb in the blue spectral region and therefore the electrodes appear yellowish colored. Already with an antimony content of 2%, this effect does not appear anymore, which can be attributed to the better conductivity of the doped nanoparticles, which allows the extraction of all injected electrons after negative polarization. However, compared to undoped SnO_2_, the ATO 2 electrodes are slightly less transparent at higher wavelengths due to the presence of free electrons, which compensate for the introduced Sb^5+^ ions. With increasing doping concentration, this effect becomes even more pronounced, and the overall transparency drops. In fact, while the ATO 2 electrodes display an average transmission of 94.5%, this value is reduced to 79% in the case of the ATO 15 nanoparticle layers. From these experiments, it therefore can be concluded that a low antimony doping concentration of 2% ensures sufficient conductivity in the desired potential range but still offers high transparency over the visible spectral region.

**Fig. 4:**
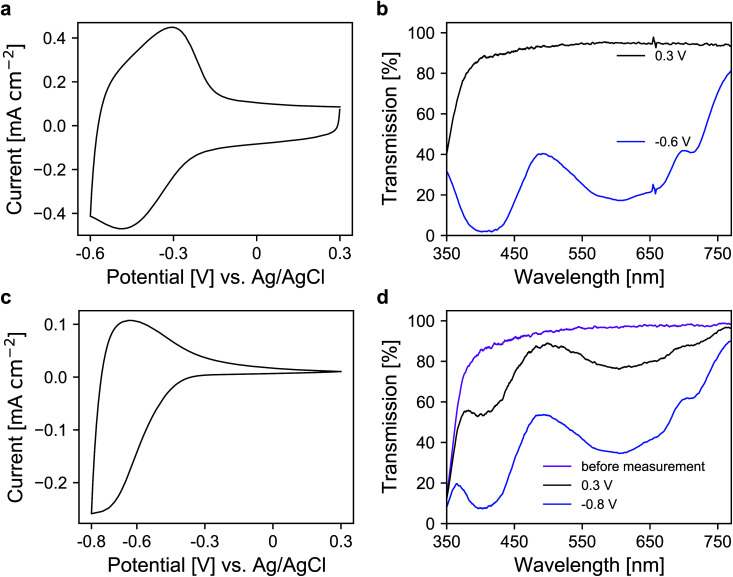
CVs of viologen modified (a) ATO and (c) TiO_2_ mesoporous electrodes measured with TBAP/PC as the electrolyte with a scan rate of 20 mV s^−1^. UV/VIS transmission spectra of the viologen modified (b) ATO and (d) TiO_2_ electrodes in colored and bleached states. Optical reference: an electrolyte-filled cell with blank FTO glass.

In the next step, a redox-active dye, namely 1-(4-cyanophenyl)-1′-(2-phosphonoethyl)-4,4′-bipyridin-1-ium (viologen), was anchored to the mesoporous nanoparticle layers *via* chemisorption. For the coloration of the dye, electrons are transported through the metal oxide scaffold to the organic molecules and *vice versa* for decoloration. The antimony doping concentration of the ATO nanoparticles was 2% and TBAP/PC was utilized as the electrolyte. A CV of a modified electrode is shown in Fig. 4a, which displays a set of reversible peaks associated with the reduction/oxidation of the viologen dye. Thus, the intrinsic conductivity of ATO still allows unrestricted switching of the dye despite the absence of lithium within the electrolyte. Since the reduction of the dye results in its coloration, the corresponding UV/VIS spectra were recorded as shown in Fig. 4b. In the colored state (−0.6 V *vs.* Ag/AgCl), the transparency of the electrode is reduced to 25%. At +0.3 V (transparent state), the modified electrode reaches an average transmission of around 91%. For comparison, we also prepared mesoporous electrodes of TiO_2_, which is the standard scaffold in combination with viologens as outlined in the Introduction. Here, a commercial nanoparticle paste was used. The layers had a thickness of approx. 3.2 μm, comparable to the ATO layers, and also displayed a mesoporous structure as shown in Fig. S3 (ESI†). After modification with the viologen, CV measurements of these electrodes were performed also with TBAP/PC as the electrolyte. As shown in Fig. 4c, the behavior of the viologen/TiO_2_ electrode displays distinct differences when compared to the viologen/ATO electrodes. While the reduction of the dye remains possible, the corresponding peak in the CV is shifted towards more negative potentials. Moreover, only a small oxidation peak is observed, which indicates electron trapping within the mesoporous layer. This behavior is related to the flat band potential of TiO_2_, which is known to be shifted towards negative potentials in the absence of cations like lithium.^23^ From the CV of a bare TiO_2_ electrode displayed in Fig. S4 (ESI†), it is evident that the TiO_2_ layer only shows considerable electrochemical activity at negative potentials, whereas the doped ATO electrodes provide conductivity even at positive potentials as discussed above. The restricted oxidation of the viologen with TiO_2_ as the scaffold is also evident from the UV/VIS spectra shown in Fig. 4d. Before the measurement, the electrode displayed an average transmission of 91.2%, which is reduced to 39.4% when a potential of −0.8 V *vs.* Ag/AgCl is applied. While decoloration remains possible to some degree, the process takes a considerably longer time compared to the viologen/ATO system. After one minute of polarization at +0.3 V, the transmission of the viologen/TiO_2_ electrode reaches only 75.8%. Therefore, these results demonstrate that mesoporous ATO electrodes in combination with viologens are more suitable compared to TiO_2_ when non-intercalating electrolytes such as TBAP are used.

**Fig. 5:**
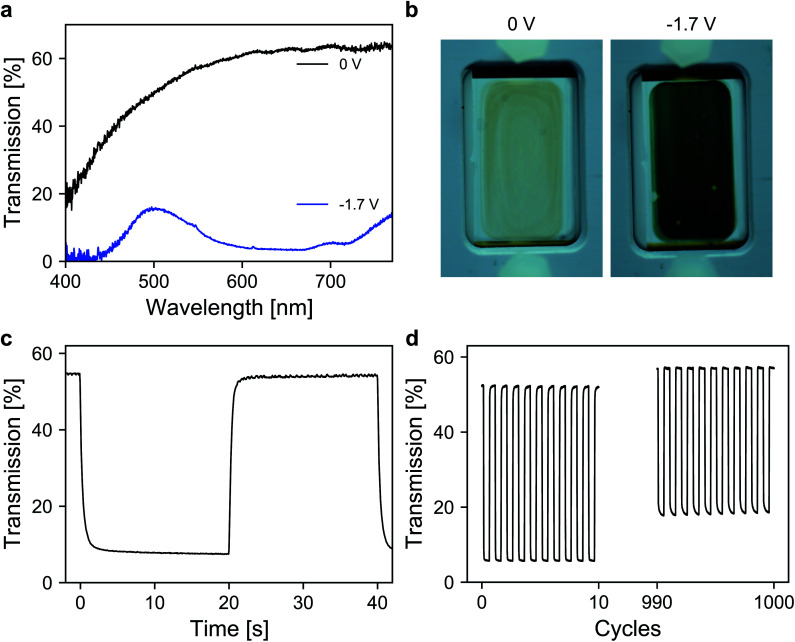
(a) Transmission spectra of the device at 0 V and −1.7 V. (b) Images of the device in the bleached (left) and colored (right) states. (c) Time depended transmission change for one switching cycle. (d) Transmission change over 1000 (de)coloration cycles.

Finally, the performance of the dye-anchored electrodes was further investigated in a sealed two-electrode device. For both electrodes, 3 μm mesoporous ATO nanoparticle layers, both doped with 2% Sb, were used as scaffolds. The working electrode was again modified with the aforementioned viologen. In the case of the counter electrode, a triarylamine based electrochromic dye, namely [4-(diphenylamino)benzyl]phosphonic acid (TAA), was used. TAA is an anodically coloring dye that undergoes oxidation on the counter electrode while the viologen is reduced on the working electrode, which allows fast switching of the device. Furthermore, the oxidation of TAA is also associated with a coloration that contributes to the overall contrast of the device. Detailed information about the ATO/TAA combination has been previously reported.^35^ A scheme of the device setup as well as the structures of the dye molecules is shown in Fig. S5 (ESI†). The electrolyte was again a 1 M solution of TBAP in PC. The transmission spectra of the device are displayed in Fig. 5a. In the colored state (−1.7 V), the transmission is reduced to 7% (average transmission over the visible range) due to the combined absorption of both dyes. The transmission value in the bleached state (0 V) reaches 62.4% (at 650 nm). The corresponding images of the device in both the uncolored and the colored state are presented in Fig. 5b. Another important characteristic of an electrochromic device is the switching time. Thus, time-series measurements for one reversible potential step were recorded (Fig. 5c). The 1/*e* switching times for coloration and decoloration are 0.3 s and 0.4 s, respectively. Considering the dense morphology of the ATO layers in combination with the large cation of the electrolyte, achieving such fast switching times is remarkable. Finally, the long-term performance of the device was investigated. For this purpose, the device was subjected to 1000 switching cycles, while monitoring the transmission as displayed in Fig. 5d. The device exhibits satisfactory stability over prolonged cycling since the electrochromic contrast only drops to 84% of its initial value after 1000 switching cycles.

## 3 Conclusions

In summary, we have presented mesoporous ATO electrodes with a low antimony concentration and small particle size as a promising material for combination with cathodically coloring dyes in electrochromic devices. Comparing the spectroelectrochemical response between LiClO_4_/PC and TBAP/PC as electrolytes showed that the utilization of the latter is beneficial with ATO. In this case, conductivity over the desired potential range is retained without impairment of the transparency, which occurs with the lithium-containing electrolyte due to intercalation after negative polarization. Furthermore, an antimony doping concentration of 2% was chosen as optimum as it enables the best compromise between electrical conductivity and optical transparency compared to undoped particles as well as higher antimony concentrations. The viologen modified ATO electrode, in combination with TBAP as the electrolyte, exhibited fully reversible (de)coloration of the dye compared to a similar electrode based on TiO_2_, which displayed restricted decoloration. Finally, the viologen/ATO electrode was applied as the working electrode in a two-electrode device with a triphenylamine/ATO based counter electrode. The device allowed fast switching (below 0.5 s) and displayed good long-term stability over 1000 cycles as it retained 84% of its initial contrast.

## 4 Experimental

### ATO nanoparticle synthesis

ATO particles with an antimony doping concentration of 2% were synthesized as follows: 2 mmol potassium hexahydroxoantimonate (0.53 g, Merck) and 2 mL hydrogen peroxide solution (30%, Merck) were added to a solution of 100 mmol potassium hydroxide (5.61 g, Merck) in 400 mL water and subsequently heated at 60 °C until a clear solution was obtained. At room temperature, 98 mmol potassium hexahydroxostannate (29.3 g, Sigma Aldrich) and 250 mmol hydrochloric acid (21 mL (37%) diluted to a total of 100 mL, Fluka) were subsequently added. After heating the solution to 95 °C for 30 min, a 1 M potassium hydroxide solution was added dropwise until the solution became clear followed by further heating for 30 min. The pH of the cooled solution was adjusted to 4 by the addition of hydrochloric acid. The precipitate was centrifuged and repeatedly washed with water until the supernatant remained turbid after centrifugation. A colloidal solution in 200 mL water was obtained after the addition of 2 mL 1,8-diazabicyclo[5.4.0]undec-7-en (DBU, purchased from TCI) to the precipitate. The colloid was then loaded into a Teflon-lined autoclave (400 mL capacity) and heated to 200 °C for 5 h after purging the solution with nitrogen for 20 min. In the next step, the volume of the colloid was reduced to 50 mL on a rotary evaporator followed by dialysis (MWCO 6000–8000) for 2 days against water. Finally, all solvent was removed on a rotary evaporator yielding the ATO nanoparticle powder. In the same manner, undoped SnO_2_, as well as ATO nanoparticles with antimony concentrations of 5% and 15% were synthesized.

### Electrode preparation

Mesoporous ATO nanoparticle layers were prepared on fluorine-doped tin oxide coated glass (∼7 Ω sq^−1^, Sigma Aldrich) by the doctor blade method. Prior to doctor blading, the substrates were cleaned by repeated ultrasonication in water and acetone followed by plasma cleaning (Diener Femto). A paste was prepared by mixing 2 g of the synthesized ATO nanoparticles and 0.5 g of hydroxypropylcellulose (Alfa Aesar) in 10 mL of a 1/1 water/ethanol mixture. After doctor blading, the electrodes were heated to 450 °C with a ramp of 5 °C min^−1^ and a dwelling time of 1 h at 450 °C. The resulting films had a thickness of approx. 3.5 μm. In a similar way, mesoporous TiO_2_ electrodes (approx. 3.2 μm) were prepared from a commercial TiO_2_ paste (Ti-Nanoxide T/SP, Solaronix). For the modification of the electrodes with a redox-active dye, the mesoporous layers were immersed into a 4 mM solution of 1-(4-cyanophenyl)-1′-(2-phosphonoethyl)-4,4′-bipyridin-1-ium (Vio) in ethanol for 16 h.

### Spectroelectrochemical characterization

Bare and dye-modified electrodes were characterized in a three-electrode setup with a platinum wire counter electrode and an Ag/AgCl reference electrode. As electrolytes, we used either lithium perchlorate (Sigma Aldrich) or tetrabutylammonium perchlorate (Sigma Aldrich) in propylene carbonate (Sigma Aldrich) with 1 M concentration. A Gamry Interface 1000 potentiostat was used to perform electrochemical experiments. Optical measurements were recorded using an Avantes AvaLight-DHc light source and an Avantes AvaSpec-3648 spectrometer. The measurements were performed in an electrochemical cell with an attached cuvette, into which the electrodes were immersed. Blank FTO glass and the electrolyte-filled cuvette were set as optical references.

### Physical characterization

The nanoparticles were characterized by X-ray powder diffraction (XRD) using a Panalytical Empyrean diffractometer with Bragg–Brentano geometry, Cu K-α radiation, and a step size of 0.026° for 2*θ*. Transmission electron microscopy (TEM) images were recorded on a JEOL JEM-2100Plus with an EM-24830FLASH camera (JEOL). The cathode material was LaB_6_ operated at 200 kV. Dynamic light scattering (DLS) measurements of aqueous colloidal solutions of ATO nanoparticles after autoclave treatment were performed on a Malvern Zetasizer Nano ZSP. Scanning electron microscopy (SEM) images were obtained with a Zeiss Auriga scanning electron microscope using an in-lens detector. The acceleration voltage was 3 kV and the samples were sputtered with platinum/iridium.

### Device fabrication

In the device, indium tin oxide coated glass was used as the substrate. The device consisted of a mesoporous 3 μm thick ATO working electrode modified with Vio and a similar (3 μm) ATO counter electrode modified with [4-(diphenylamino)benzyl]-phosphonic acid (TAA). The modification of the ATO electrode with TAA was performed by the immersion of the electrode into a 4 mM solution of TAA in ethanol for 16 h. As the electrolyte, a 1 M solution of tetrabutylammonium perchlorate in propylene carbonate was used. The details of the device fabrication are reported elsewhere.^45^

## Conflicts of interest

There are no conflicts to declare.

## Supplementary Material

NA-004-D1NA00877C-s001
