# Supplementary material for: Intercalation-free, fast switching of mesoporous antimony doped tin oxide with cathodically coloring electrochromic dyes
Source: Nanoscale Adv. 2022 Mar 17;4(9):2144–52. doi: 10.1039/d1na00877c (PMC9418254; doi:10.1039/d1na00877c)
Supplement: NA-004-D1NA00877C-s001 [file NA-004-D1NA00877C-s001.pdf]

Electronic Supplementary Material (ESI) for Nanoscale Advances.
This journal is © The Royal Society of Chemistry 2022

Supporting Information

Intercalation-free, Fast Switching of Mesoporous Antimony Doped Tin Oxide with Cathodically Coloring Electrochromic Dyes

Jonas Klein, Alexander Hein, Ellen Bold, Fatih Alarslan, Egbert Oesterschulze, Markus Haase

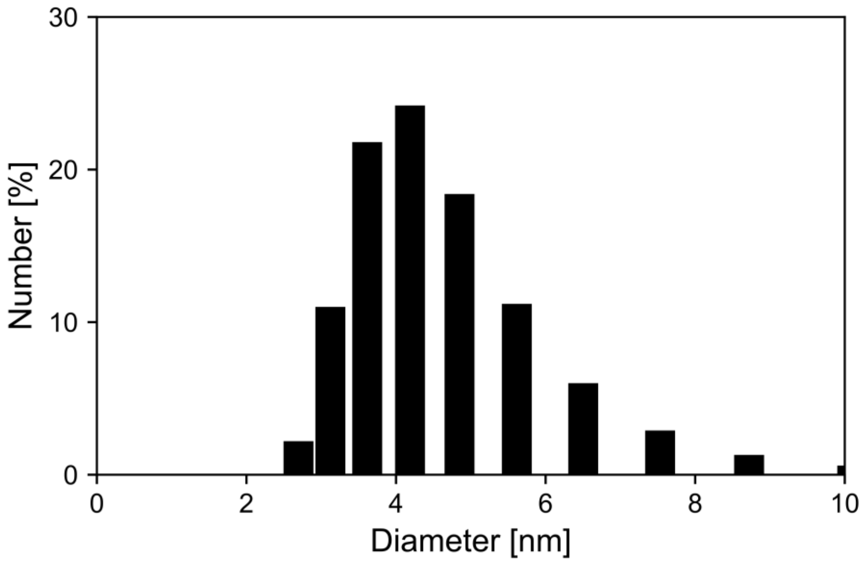

Figure S1. Dynamic light scattering measurement of colloidal ATO (2 % Sb) nanoparticles after synthesis and autoclave treatment measured in water.

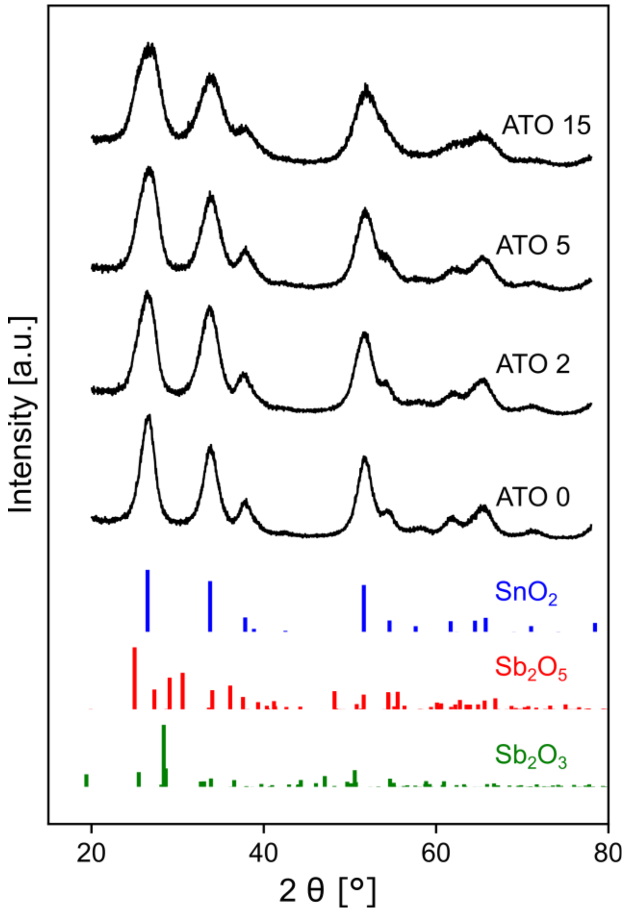

Figure S2. X-ray powder diffractogram of ATO particles after autoclave treatment with antimony contents of 0 % (ATO 0), 2 % (ATO 2), 5 % (ATO 5) and 15 % (ATO 15) along with reference spectra of SnO_2 (ICSD: 39177, blue), Sb_2O_5 (ICSD: 8050, red) and Sb_2O_3 (ICSD: 2033, green).

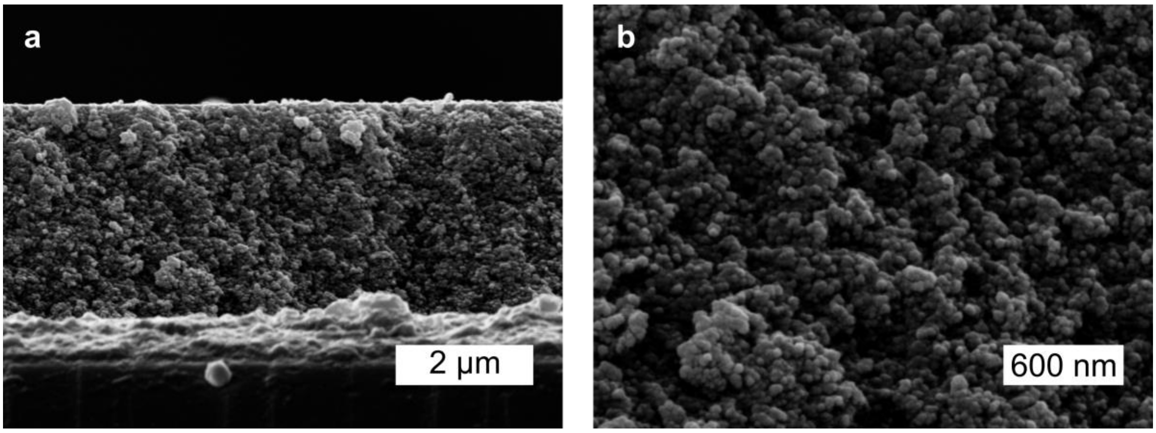

Figure S3. a) Cross section SEM of TiO_2 layers on FTO. b) Magnified cross section view displaying the mesoporous structure of the nanoparticle layers.

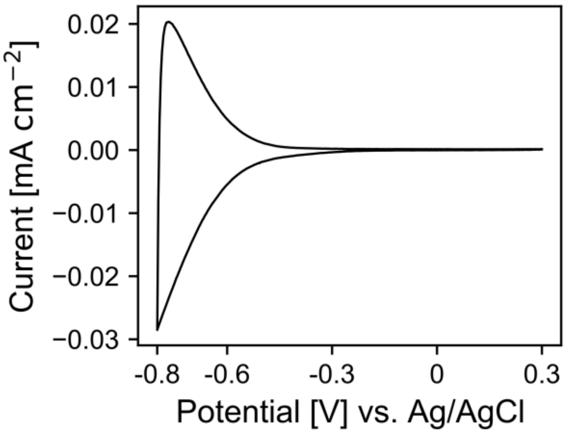

Figure S4. CV of a TiO_2 mesoporous electrode layers measured in TBAP/PC with a scan rate of 20 mV s^{-1}.

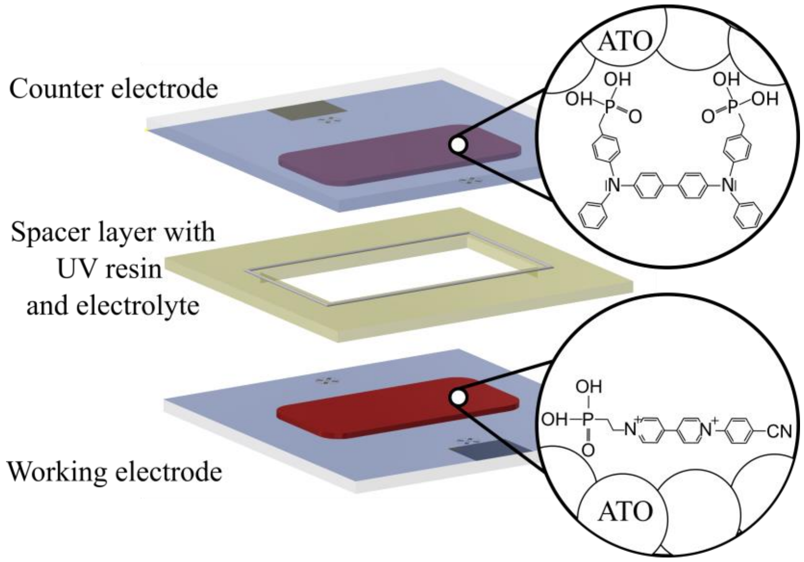

Figure S5. Exploded view of constructed device with the chemical structure of the EC materials given in the enlarged schemes. The figure shows the structured gold layers with the alignment marks, the ITO coating (light blue) as well as the rectangular-shaped doctor bladed nanoparticle layers. A spacer layer (yellow, Ordyl) is structured to form a rectangular cavity for the electrolyte. For mechanical stability and hermetic encapsulation, we dispense UV resin (grey) on the spacer in the shape of a frame. Before bonding, electrolyte is dispensed in the rectangular cavity. Then the whole stack is pressed together and illuminated with UV light to cure the resin.
